# Anti-oxidative and anti-inflammatory activities of the ethanol extract of edible flower from *Chimonanthus praecox*

**DOI:** 10.3389/fphar.2022.1004520

**Published:** 2022-09-27

**Authors:** Yuan Kang, Zhuangzhuang Liu, Wenjing Li, Ximeng Li, Xiaoyu Zhang, Zhenlu Xu, Yuan Gao, Yun Qi

**Affiliations:** Institute of Medicinal Plant Development, Chinese Academy of Medical Sciences and Peking Union Medical College, Beijing, China

**Keywords:** wintersweet flower, inflammation, LPS, NF-κB, endotoxemia, RAW264.7 macrophages, ROS, NADPH

## Abstract

Chimonanthi Praecocis Flos, namely wintersweet flower, is the edible flower or flower bud of *Chimonanthus praecox* (L.) Link which is a deciduous shrub plant originated from China and is widely cultivated as a garden or ornamental plant all over the world. However, few studies focused on its anti-inflammatory property. In the present study, we explored the anti-inflammatory and anti-oxidative activities of ethanol extract of Chimonanthi Praecocis Flos (CPE) which contained 7.980% ± 0.176% total flavonoids and 1.461% ± 0.041% total alkaloids. In LPS-stimulated RAW264.7 macrophages, CPE significantly decreased the production of NO and prostaglandin E_2_ (PGE_2_) through reducing the expressions of their synthases—inducible nitric oxide synthase (iNOS) and cyclooxygenase-2 (COX-2). It also suppressed the transcription and translation of pro-inflammatory cytokines interleukin-1β (IL-1β) and interleukin-6 (IL-6). Further research revealed that CPE impeded the phosphorylation and degradation of IκBα, thus restraining the nuclear translocation of p65, and consequently dampening NF-κB signaling. In endotoxemia mice, several pro-inflammatory cytokines in serum were also decreased after CPE treatment. Besides anti-inflammatory activity, anti-oxidative activity is another important capacity of wintersweet flower. Indeed, CPE reduced LPS-elevated intracellular total reactive oxygen species (ROS) level by weakening NADPH oxidase activity in cell system. Moreover, it directly scavenged DPPH radical and superoxide anion, and exerted ferric reducing ability in cell-free system. Our findings demonstrate that wintersweet flower can be used as a beneficial natural product or an additive by virtue of its anti-oxidative and anti-inflammatory properties.

## 1 Introduction

Macrophages, existing in almost all tissues, are essential effector cells of the innate immune system for resisting the invasion of pathogenic microorganisms and maintaining immune homeostasis. Toll-like receptor 4 (TLR4) on the surface of macrophages can recognize and bind to lipopolysaccharide (LPS), a major surface component of Gram-negative bacteria (Poltorak et al., 1998). Once engaged by LPS, TLR4 dimerization triggers the recruitment of myeloid differentiation primary response 88 (MyD88), thereby transmitting a series of downstream signaling cascades that lead to the activation of nuclear factor κB (NF-κB) pathway (Lu et al., 2008). In the resting state, NF-κB dimers (p50/p65) are sequestered in the cytoplasm by the inhibitors of NF-κB (IκBs). Upon stimulation, the IκB kinase (IKK) phosphorylates IκBα, which leads to the ubiquitination and degradation of IκBα and allows NF-κB to translocate from cytoplasm to nucleus (Peng et al., 2020). As a result, released NF-κB binds to specific DNA elements and regulates downstream transcriptions of inflammatory genes, such as inducible nitric oxide synthase (iNOS), cyclooxygenase-2 (COX-2), interleukin-6 (IL-6), and interleukin-1β (IL-1β) (Tak and Firestein, 2001). Although moderate inflammation is indispensable for blocking the invasion of pathogens, overactivated inflammatory responses may contribute to tissue damage and organ dysfunction.

Oxidative stress, which is defined as an imbalance between the generation of oxidants and antioxidant defenses, is another important participant in the development and progression of numerous diseases (Zucker et al., 2014). At physiological concentrations, reactive species [reactive oxygen species (ROS) and reactive nitrogen species (RNS)], which can be produced continuously and reduced readily by the antioxidant defense system, function in many cellular activities (Van der Pol et al., 2019). In oxidative stress, non-physiological production of reactive species not only can directly oxidize biomacromolecules (e.g., membrane lipids, proteins and nucleic acids) thus leading to cell damage and death, but also can disturb various redox signaling and affect multiple pathophysiological processes, including inflammation (Forman and Zhang, 2021). Hence, effective suppression of inflammation and oxidative stress is considered to be a crucial clinical strategy for the treatment of many diseases.

**FIGURE 1:**
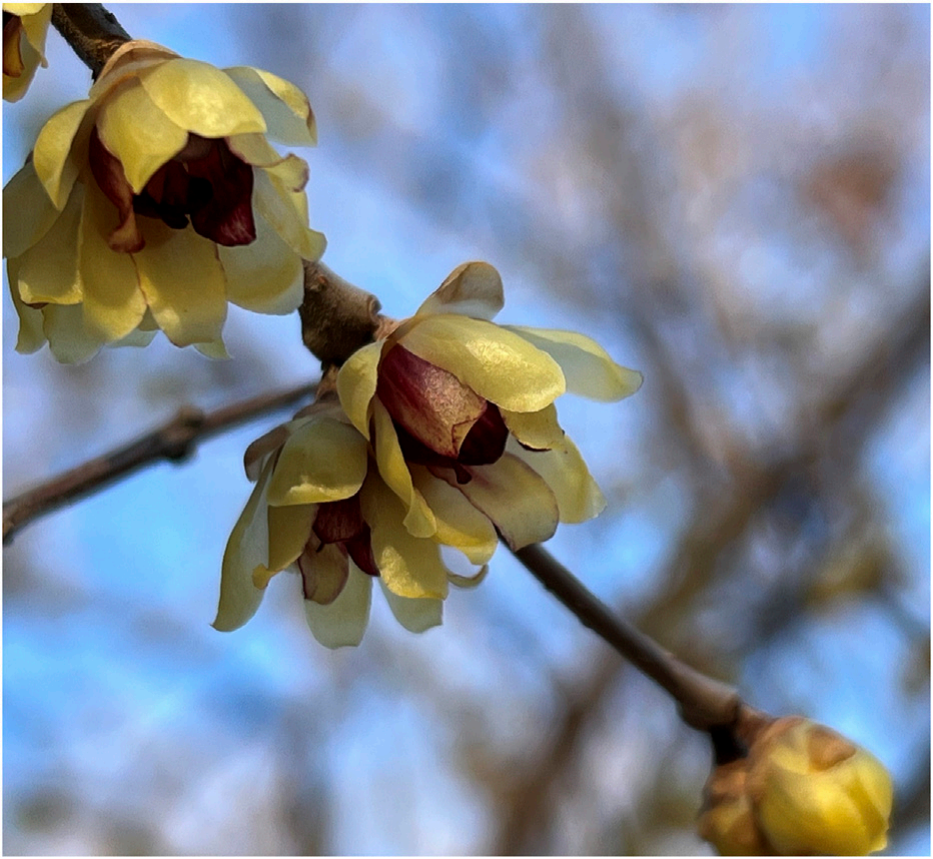
The fresh flowers of *Chimonanthus praecox* (L.) Link.

Chimonanthi Praecocis Flos, commonly known as wintersweet flower (La-Mei-Hua in Chinese), is the flower or flower bud of *Chimonanthus praecox* (L.) Link (Figure 1) which is a deciduous shrub plant of the Calycanthaceae family originated from China. Owing to the unique flowering time, long blooming period, and pleasant fragrance, *C. praecox* (L.) Link is widely cultivated as a garden or ornamental plant in the world (Li et al., 2016). The edibility of Chimonanthi Praecocis Flos was first recorded in the *Jiuhuang Bencao* (A.D. 1406). And the subsequent *Bencao Gangmu* (A.D. 1449) recorded its effects on relieving summer-heat and slaking thirst which may be a reason for using it as tea drinks in Chinese folk (Hu et al., 2019). In addition, it also has been used as dishes, desserts, and wine (Cheng et al., 2007; Yang et al., 2013). Previous phytochemical studies have reported that wintersweet flower contains a variety of bioactive substances, such as volatile oils (Deng et al., 2004), alkaloids (Zhang et al., 2009), flavonoids (Yang et al., 2018), and phenolic acids (Li et al., 2016). Modern pharmacological studies reported that the essential oil and semi-volatile fractions of wintersweet flower possessed antimicrobial and anti-oxidative activities (Lv et al., 2012), while its methanol extract inhibited melanogenesis (Morikawa et al., 2014). The water extract of wintersweet flower could significantly enhance the phagocytic activity of macrophage and humoral immune function of mice (Shu et al., 2019). The root of *C. praecox* (L.) Link is yet used for treating rheumatoid arthritis (Li and Qian, 2016), suggesting that wintersweet flower may also possess an anti-inflammatory effect. However, the anti-oxidative and anti-inflammatory properties of wintersweet flower remain poorly characterized. In this study, we not only investigated the anti-oxidative activity of the ethanol extract of Chimonanthi Praecocis Flos (CPE), but also explored its anti-inflammatory actions and mechanisms.

## 2 Materials and methods

### 2.1 Reagents

Calycanthine and chimonanthine were from Push Bio-Technology Co., Ltd. (Chengdu, Sichuan, China). Rutin and quercetin were from China Institute of Food and Drug Verification (Beijing, China). Recombinant mouse M-CSF protein was purchased from Sino Biological Inc. (Beijing, China). MTS was purchased from Promega Co., Ltd. (Madison, WI, United States). LPS, L-NAME, DPPH, lucigenin and apocynin were from Sigma-Aldrich (St. Louis, MO, United States). Griess reagent NO assay kit, pNFκB-TA-luc and pAP1-TA-luc reporter plasmids, luciferase assay system, and monoclonal antibodies against IκBα and p-IκBα (Ser32) were obtained from Beyotime Biotechnology (Shanghai, China). Mouse IL-6, TNF-α, and MCP-1 ELISA kits were from BioLegend Inc. (San Diego, CA, United States). Mouse IL-1β ELISA kit was from Excell Biotech Co., Ltd. (Taicang, Jiangsu, China). PGE_2_ high sensitivity ELISA kit was purchased from Enzo Life Sciences, Inc. (Farmingdale, NY, United States). Celecoxib was obtained from Solarbio Co., Ltd. (Beijing, China). Nigericin (sodium salt) was from MedChemExpress (Shanghai, China). Antibodies against iNOS and NF-κB p65 were obtained from Cell Signaling Technology (Danvers, CO, United States). M-MuLV first strand cDNA Synthesis kit was from Sangon Biotech Co., Ltd. (Shanghai, China). Antibodies against GAPDH, Histone H3, and HRP goat anti-mouse IgG were from ABclonal Biotech Inc. (Wuhan, Hubei, China). L-012 was from Wako Pure Chemical Corp. (Osaka, Japan).

### 2.2 Cells and animals

RAW264.7 macrophages were from the American Type Culture Collection (ATCC, Rockville, MD, United States) and cultured in DMEM supplemented with 10% FBS in a humidified incubator with 5.0% CO_2_ at 37°C. Bone marrow-derived macrophages (BMDMs) were isolated from male C57BL/6 mice and then differentiated with M-CSF (100 ng/ml) for 7 days as previously described (Han et al., 2020).

The SPF male C57BL/6 mice (18–20 g) were purchased from Vital River Experimental Animal Services [Beijing, China; license number: SCXK (Beijing) 2021-0006]. All experiments were approved by the Care and Welfare of Laboratory Animals Committee of the Institute of Medicinal Plant Development of Chinese Academy of Medical Sciences and Peking Union Medical College, complied with the ARRIVE guidelines, and were performed in compliance with the Guide for the Care and Use of Laboratory Animals (8th edition). The anesthetic drug isoflurane and all other necessary measures were used to relieve the suffering of animals during the experiments.

### 2.3 Preparation of CPE

Wintersweet flower was collected in the senescing flower stage from Haidian District of Beijing in March 2021 and identified by Prof. Yulin Lin (Institute of Medicinal Plant Development, Chinese Academy of Medical Sciences and Peking Union Medical College, Beijing, China) (Figure 1). The voucher specimen (BJ-2021-ML-331) was deposited in the Institute of Medicinal Plant Development, Chinese Academy of Medical Sciences & Peking Union Medical College. The shade-dried flowers were soaked in 85% ethanol (1:25, w/v) overnight and subsequently extracted twice by refluxing for 2 h each time. The obtained extract (CPE) was filtered and then concentrated *via* rotary evaporation at 50°C. The residue was collected and dried to a constant weight. The yield was 33.67% (w/w).

### 2.4 HPLC conditions

The components of CPE were analyzed by HPLC according to previous protocol with slight modifications (Shu et al., 2017; Zhang et al., 2021). HPLC analysis was carried out with an Essentia LC-15C HPLC system (Shimadzu, Japan) equipped with a UV/Vis detector (Shimadzu, Japan) and a reversed-phase Syncronis™ C18 column (250 mm × 4.6 mm, 5 μm; Thermo Fisher Scientific, United States). The flow rate was 1.0 ml/min, and the column temperature was at room temperature. The gradient elution programs for alkaloids and flavonoids were shown in Tables 1, 2.

**TABLE 1:** Gradient elution program for alkaloids (detection wavelength: 239 nm).

Time (min)	Mobile phases	
	A (0.01 mol/L KH_2_PO_4,_ pH 3.5)	B (methanol)
0–30	90%–56%	10%–44%

**TABLE 2:** Gradient elution program for flavonoids (detection wavelength: 360 nm).

Time (min)	Mobile phases	
	A (0.1% formic acid)	B (acetonitrile)
0–5	88%	12%
5–10	88%–75%	12%–25%
10–30	75%–25%	25%–75%

### 2.5 Determination of total flavonoids and total alkaloids

Determination of total flavonoids was performed according to the NaNO_2_-Al(NO_3_)_3_-NaOH colorimetric method (Sun et al., 2020). The samples (1.2 ml, dissolved in 40% ethanol) were placed in glass test tubes, and then 200 μl of 5% NaNO_2_ solution was added before the mixture was shaken and incubated at 25°C for 6 min. Next, 200 μl of 10% Al (NO_3_)_3_ solution was added. After a further 6 min, 2 ml of 4.3% NaOH solution and 1.4 ml of distilled water were then added followed by 15 min of reaction. The optical density (OD) value was read at 492 nm and rutin was used as a standard.

The total alkaloids content was determined by the acid dye colorimetric method (Guo et al., 2019). The samples (5 ml, dissolved in chloroform) were placed in glass test tubes, and then 2.5 ml of distilled water and 1 ml of bromocresol green buffer (0.05% bromocresol green in 1% KH_2_PO_4_ and 12 mM NaOH) were added. Subsequently, the tubes were sealed with rubber stoppers and vigorously shaken for 1 min. 15 min later, the samples were centrifuged at 1,500 *g* for 10 min and 200 μl of the lower chloroform layer was obtained to measure the OD value at 414 nm. Chimonanthine was used as a standard.

### 2.6 Cytotoxicity assay

The cell viability assay was carried out by MTS method. RAW264.7 macrophages were plated in 96-well plates at a density of 4 × 10^5^ cells per well and incubated with CPE at different concentrations for 22 h. Then, MTS (2 mg/ml in DPBS) and PMS (0.92 mg/ml in DPBS) in a ratio of 20:1 were added (20 μl/well) according to previous description (Goodwin et al., 1995). 2 h later, the OD value at 492 nm was determined.

### 2.7 Measurement of anti-oxidative capacity in cell-free systems

Ferric reducing ability of CPE was determined as previously described (Wang et al., 2008). Trolox, a hydrophilic analog of vitamin E, was used as a standard.

DPPH radical scavenging activity was determined as we previously described (Wang et al., 2008). Trolox at 50 μM was used as a positive control.

Superoxide anion scavenging activity was determined as we previously described (Wang et al., 2008). Rutin at 20 μM was used as a positive control.

### 2.8 Measurement of anti-oxidative capacity in cell system

#### 2.8.1 Measurement of intracellular ROS

RAW264.7 macrophages were plated at a density of 4 × 10^5^ cells per well in a 96-well plate and incubated with LPS (8 ng/ml) with or without CPE for 24 h. After washing the cells twice with D-Hank’s, 100 μl of L-012 probe (100 μM) was added, followed by incubation at 37°C for 30 min. The chemiluminescence was detected using a Perkin Elmer 2450 MicroPlate Counter, and the chemiluminescence in the normal control group was normalized to 100% (Imada et al., 1999). Apocynin (150 μM) was used as a positive control.

#### 2.8.2 Determination of intracellular NADPH oxidase activity

RAW264.7 macrophages were plated in a 6-well plate at a density of 1 × 10^7^ cells per well. After treating with LPS (8 ng/ml) with or without CPE for 6 h, the cells were washed twice with PBS and collected. Subsequently, cells were sonicated on ice in a buffer containing 1 mM EDTA, protease inhibitor cocktail, and 50 mM KH_2_PO_4_ (pH 7.0). Cell lysate was collected after centrifuging at 13,000 *g* for 5 min at 4°C, and the protein concentration of supernatant was unified by BCA protein assay. In a 96-well white plate, 20 μl of supernatant was incubated with 80 μl of lucigenin for 5 min at 25°C and protected from light. The chemiluminescence was detected immediately after addition of 100 μl of NADPH (1 mM in 50 mM KH_2_PO_4_, pH 7.0) (Sarna et al., 2010). Apocynin (150 μM) was used as a positive control.

#### 2.8.3 Measurement of mtROS

RAW264.7 macrophages were plated at a density of 2 × 10^6^ cells per well in a 12-well plate and incubated with LPS (8 ng/ml) in the absence or presence of CPE for 8 h. After removing the supernatant, 300 μl of MitoSOX Red (5 μM) was added, followed by incubation at 37°C for 30 min. The cells were scraped off with a cell scraper and transferred to a blank 96-well plate after washing twice with D-Hank’s. The fluorescence was read at 510 nm excitation and 580 nm emission by a fluorescence microplate analyzer (Thermo Scientific Fluoroskan Ascent FL, United States).

### 2.9 Measurement of supernatant pro-inflammatory mediators

RAW264.7 macrophages were plated in a 96-well plate at a density of 4 × 10^5^ cells per well and treated with LPS (8 ng/ml) in the absence or presence of CPE for 24 h. NO level in the medium was then determined by Griess reaction (Li et al., 2012). Supernatant PGE_2_, IL-6, and IL-1β levels were then detected by ELISA according to their manufacturers’ protocols, respectively. Their concentrations were calculated against their respective standard curves. L-NAME, celecoxib, and dexamethasone were used as the positive controls.

### 2.10 Measurement of the activity of iNOS

The iNOS activity was determined as reported previously with some modifications (Mitchell et al., 1993). RAW264.7 macrophages were plated in a 25 cm^2^ culture flask and incubated with LPS at 8 ng/ml to induce iNOS. LPS was removed 12 h later by washing the cells 3 times. And then the cells were harvested and plated in a 96-well plate at a density of 4 × 10^5^ cells per well. The cells were cultured in the presence or absence of CPE at different concentrations for another 12 h. Supernatant NO was measured to represent the activity of iNOS. L-NAME was used as the positive control.

### 2.11 NLRP3 inflammasome activation assay

To study the activation of NLRP3 inflammasome, the method reported previously was adopted with some modifications (Qin et al., 2021). BMDMs were plated in a 48-well plate at a density of 8 × 10^5^ cells per well. 12 h later, the cells were primed with LPS at 8 ng/ml for an additional 12 h. LPS was then removed, and the cells were cultured in the presence or absence of CPE at different concentrations for 30 min. Nigericin (10 µM) was subsequently added to activate NLRP3 inflammasome. 45 min later, the level of supernatant IL-1β, which is positively correlated with the activity of NLRP3 inflammasome, was determined.

### 2.12 Luciferase reporter gene assay

RAW264.7 macrophages transfected with pNFκB-TA-luc or pAP1-TA-luc plasmid were plated in 24-well plates at a density of 2 × 10^6^ cells per well and treated with LPS (8 ng/ml) with or without different concentrations of CPE for 4 h. The cells were then lysed for the luciferase activity assay according to the manufacturer’s protocol.

### 2.13 RNA extraction and RT-qPCR

RNA extraction and RT-qPCR assay were carried out as previously described (Zhang et al., 2021). In brief, RAW264.7 macrophages were plated at a density of 1 × 10^7^ cells per well in 6-well plates. The cells were treated with LPS (8 ng/ml) in the presence or absence of CPE for 4 h. Total mRNA was then extracted. The levels of iNOS, IL-6, and COX-2 mRNA were normalized to GAPDH. The primers’ sequences were shown in Table 3.

**TABLE 3:** Primers used in the RT-qPCR analysis.

Gene	Primer sequences
iNOS	F: 5′-CTC AGC CCA ACA ATA CAA G-3′
	R: 5′-CTA CAG TTC CGA GCG TCA-3′
COX-2	F: 5′-CAT CCC CTT CCT GCG AAG TT-3′
	R: 5′-CAT GGG AGT TGG GCA GTC AT-3′
IL-6	F: 5′-CTG CAA GAG ACT TCC ATC CAG-3′
	R: 5′-AGT GGT ATA GAC AGG TCT GTT GG-3′
IL-1β	F: 5′-GCA ACT GTT CCT GAA CTC AAC T-3′
	R: 5′-ACT TTT TGG GGT CCG TCA ACT -3′
GAPDH	F: 5′-GGT TGT CTC CTG CGA CTT CA-3′
	R: 5′-TGG TCC AGG GTT TCT TAC TCC-3′

F, forward; R, reverse.

### 2.14 Western blot

RAW264.7 macrophages were plated in 6-well plates (1 × 10^7^ cells per well) and treated with LPS (8 ng/ml) with or without CPE for different times. The cells were then collected and the total or cytosolic or nuclear proteins were extracted. Then, quantified proteins (30 μg per lane) were separated by SDS-PAGE and transferred to PVDF membranes. The membranes were blocked with 5% non-fat milk in TBST for 2 h and incubated at 4°C overnight with respective primary antibodies, and then further incubated with HRP-conjugated secondary antibodies at 25°C for 1 h after washing 3 times. The blots were developed by ECL reagent and visualized using a ChemiDoc XRS^+^ imaging system (Bio-Rad, California, United States). Quantification of Western blots was performed using ImageJ software (Stuttgart, Baden-Württemberg, Germany).

### 2.15 Mouse endotoxemia model

CPE was dissolved in normal saline containing 5% DMSO and 2% Tween-80. Male C57BL/6 mice were randomly divided into five groups: normal control group, model group (5 mg/kg LPS), and CPE groups (5 mg/kg LPS + 200 mg/kg, 400 mg/kg, or 600 mg/kg CPE). The mice were intravenously injected with normal saline (normal control group) or LPS (model group and CPE groups), and then intraperitoneal administration of vehicle (normal saline containing 5% DMSO and 2% Tween-80) or CPE immediately. 2 h later, the mice were anesthetized with isoflurane, and then the blood was collected. Pro-inflammatory cytokines in the serum were detected by ELISA.

### 2.16 Statistical analysis

All experiments were repeated at least three times and results were expressed as mean ± SD from a representative experiment. Statistical analyses were carried out using GraphPad Prism version 9.2.0 (GraphPad Software, Inc., La Jolla, CA, United States). A Student’s *t*-test was used for comparing two groups, and one-way ANOVA followed by the Tukey posttest was used for comparing more than two groups. *p* < 0.05 was considered statistically significant.

## 3 Results

### 3.1 Component analysis of CPE

**FIGURE 2:**
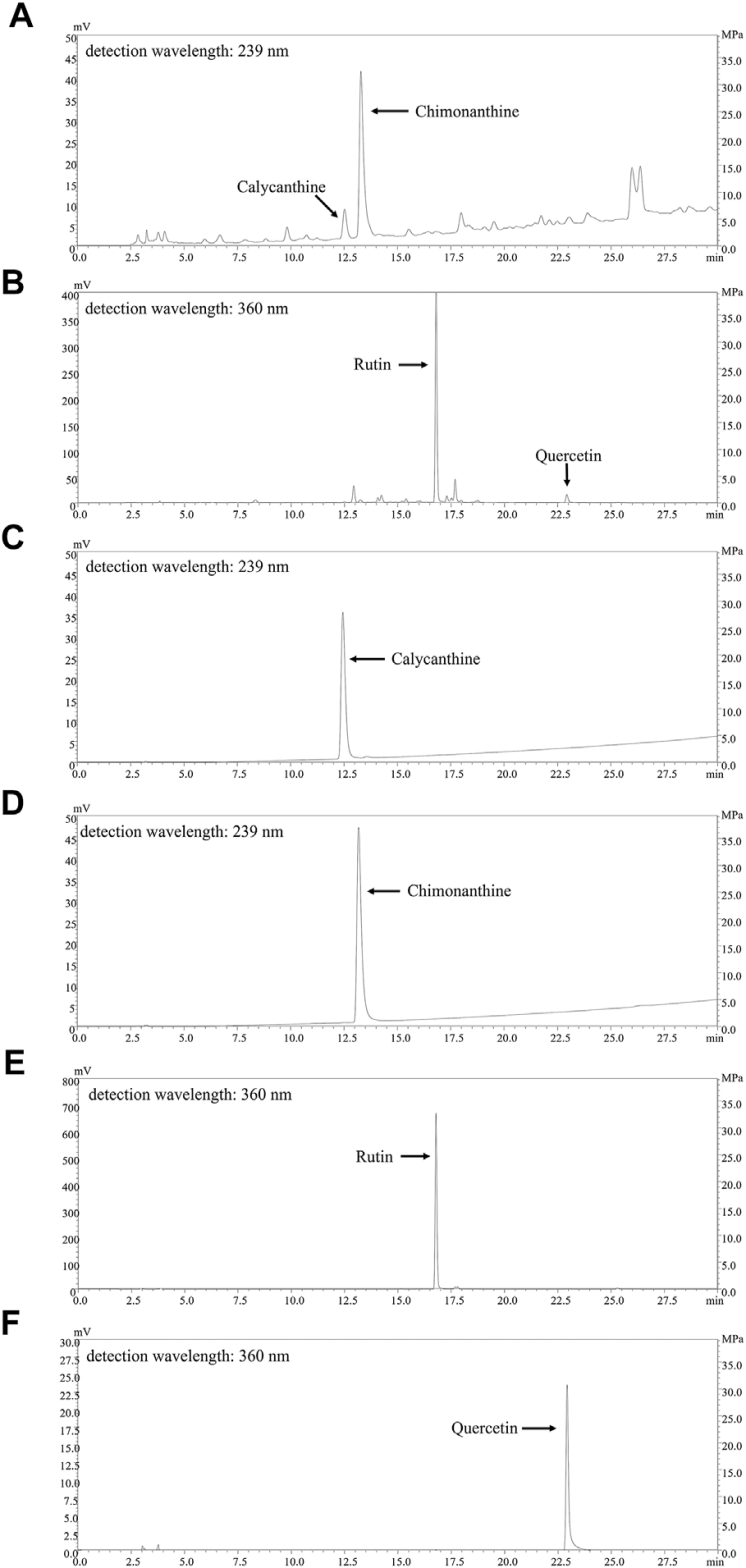
HPLC chromatograms of CPE and the responding reference substances. **(A,B)** The fingerprints of the alkaloids of CPE **(A)** and the flavonoids of CPE **(B)**. **(C–F)** The HPLC chromatograms of four reference substances: calycanthine **(C)**, chimonanthine **(D)**, rutin **(E)**, and quercetin **(F)**.

We firstly assayed total flavonoids and alkaloids in CPE and their contents were 7.980% ± 0.176% and 1.461% ± 0.041%, respectively. The fingerprints for the alkaloids and flavonoids of CPE were also determined by HPLC. As shown in Figures 2A,B, four constituents (calycanthine, chimonanthine, rutin, and quercetin) were identified by comparing their retention time with that of reference substances (Figures 2C–F). Their calculated contents in CPE were 0.047% ± 0.002%, 0.708% ± 0.015%, 4.158% ± 0.094%, and 0.128% ± 0.006%, respectively.

### 3.2 Anti-oxidative activities of CPE

#### 3.2.1 CPE exerts anti-oxidative activity in cell-free system

**FIGURE 3:**
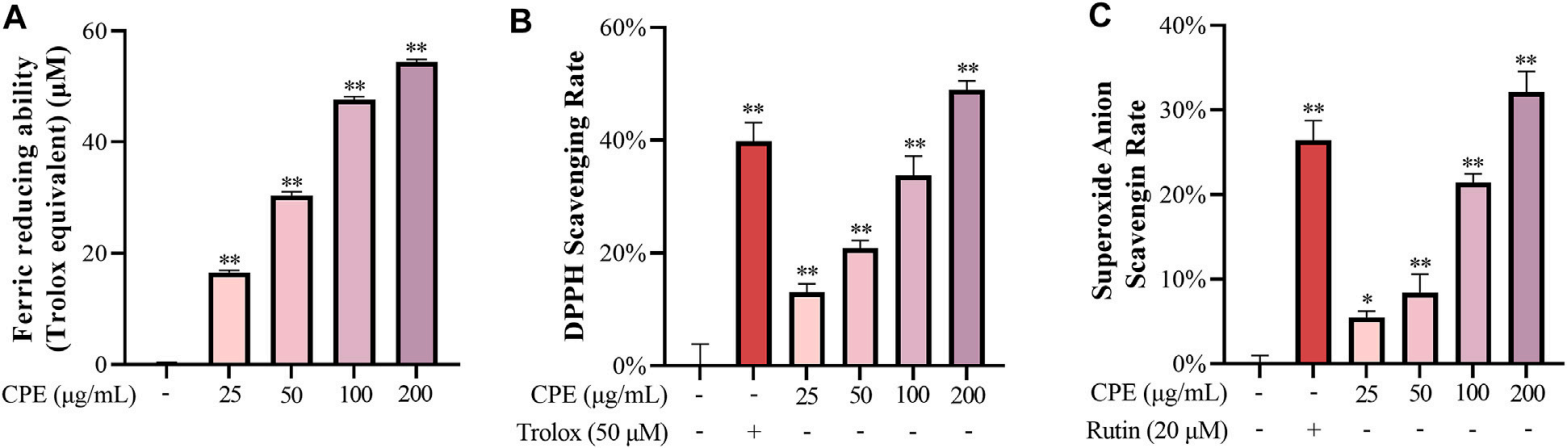
Ferric reducing ability **(A)**, DPPH scavenging activity **(B)**, and superoxide anion scavenging activity **(C)** of CPE (*n* = 3). Ferric reducing ability was expressed as Trolox (μM) equivalent antioxidant capacity. Trolox and rutin were used as positive controls for DPPH and superoxide anion scavenging activity, respectively. ^*^ *p* < 0.05 and ^**^ *p* < 0.01 vs. normal control group.

In cell-free systems, ferric reducing ability, DPPH radical scavenging activity, and superoxide anion scavenging activity of CPE were evaluated. Our data showed that CPE possessed marked ferric reducing ability (Figure 3A) and scavenging activity for DPPH radical and superoxide anion (Figures 3B,C). For measuring ferric reducing ability, 100 μg/ml of CPE was equivalent to about 47.6 μM of Trolox. And the 30% effective concentration (EC_30_) values of CPE for scavenging DPPH radical and superoxide anion were 83.8 and 176.5 μg/ml, respectively.

#### 3.2.2 CPE exerts anti-oxidative activity in cell system

**FIGURE 4:**
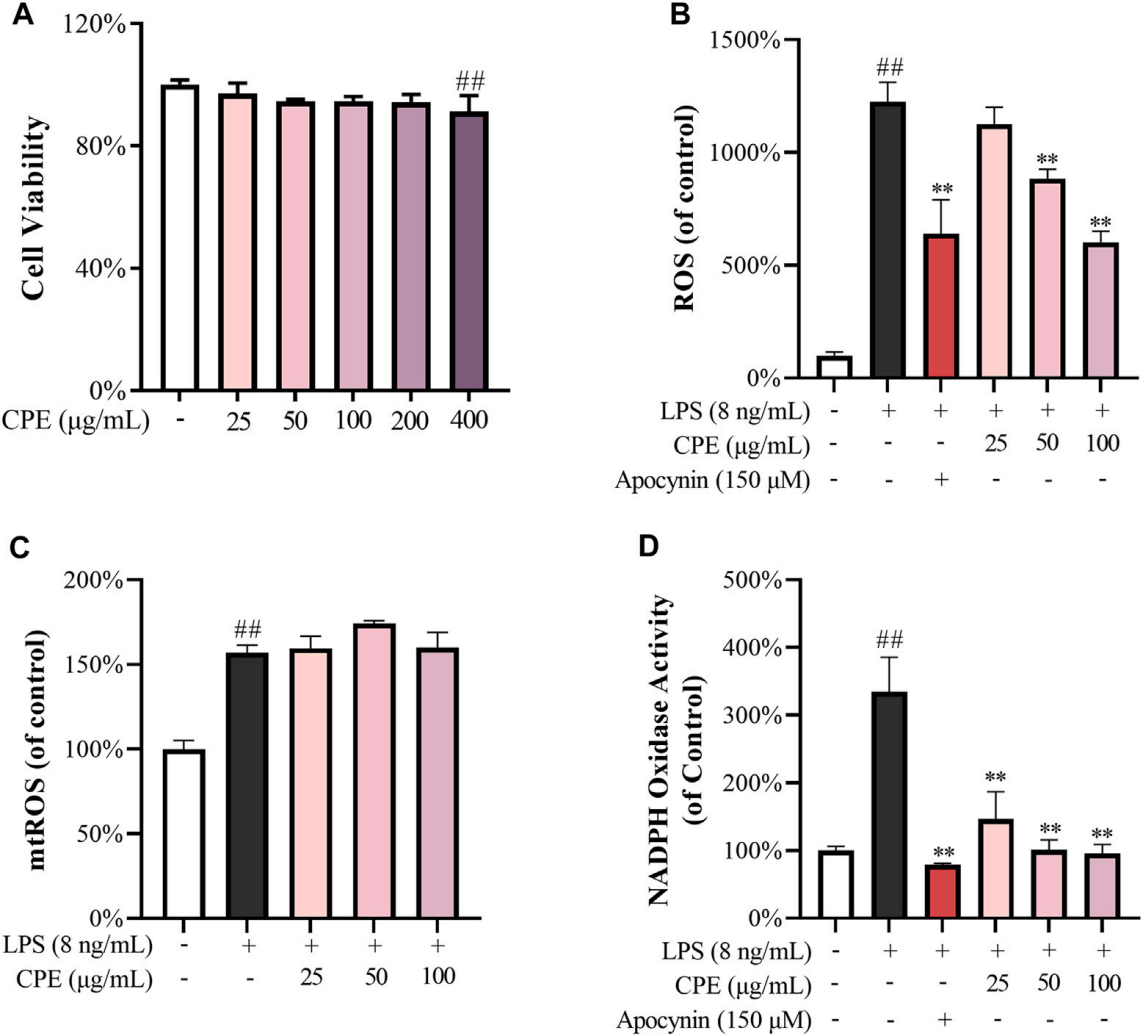
Effects of CPE on intracellular ROS (*n* = 3). **(A)** Effect of CPE on the viability of RAW264.7 macrophages. The cells were treated with CPE at the indicated concentrations for 24 h and their viability was determined by MTS assay. **(B)** Effect of CPE on intracellular ROS. RAW264.7 macrophages were exposed to LPS (8 ng/ml) and treated with or without corresponding concentrations of CPE or apocynin (positive control) for 24 h. ROS level was determined using the chemiluminescence method. **(C)** Effect of CPE on mtROS. RAW264.7 macrophages were incubated with LPS (8 ng/ml) in the absence or presence of CPE at the indicated concentrations for 8 h. The mtROS level was determined by using MitoSOX Red (5 μM) as the fluorescence probe (*λ*_ex_ 510 nm/*λ*_em_ 580 nm). **(D)** Effect of CPE on intracellular NADPH activity. RAW264.7 macrophages were treated with LPS (8 ng/ml) with or without CPE for 6 h. NADPH activity was determined using the chemiluminescence method. Apocynin was used as a positive control. ^##^ *p* < 0.01 vs. normal control group; ^**^ *p* < 0.01 vs. LPS alone group.

To rule out the non-specific inhibitory action caused by cytotoxicity, we first determined the effect of CPE on the viability of RAW264.7 macrophages. As shown in Figure 4A, CPE did not affect the cell viability up to 200 μg/ml. We next measured the anti-oxidative capacity of CPE at the non-toxic concentrations (25, 50, and 100 μg/ml) in RAW264.7 macrophages. The result showed that LPS led to approximately 12 times increase of ROS production, while CPE could significantly reduce LPS-induced ROS accumulation (Figure 4B). However, CPE did not decrease mtROS production (Figure 4C). In view of the dominant role of NADPH oxidase in the production of intracellular ROS, we next determined the effect of CPE on the activity of NADPH oxidase. As shown in Figure 4D, CPE indeed significantly inhibited LPS-activated NADPH oxidase. These results demonstrated that CPE could decrease intracellular ROS through inhibiting NADPH oxidase activity.

### 3.3 Anti-inflammatory activities of CPE

#### 3.3.1 CPE inhibits LPS-induced NO production through suppressing iNOS at the transcriptional and translational levels

**FIGURE 5:**
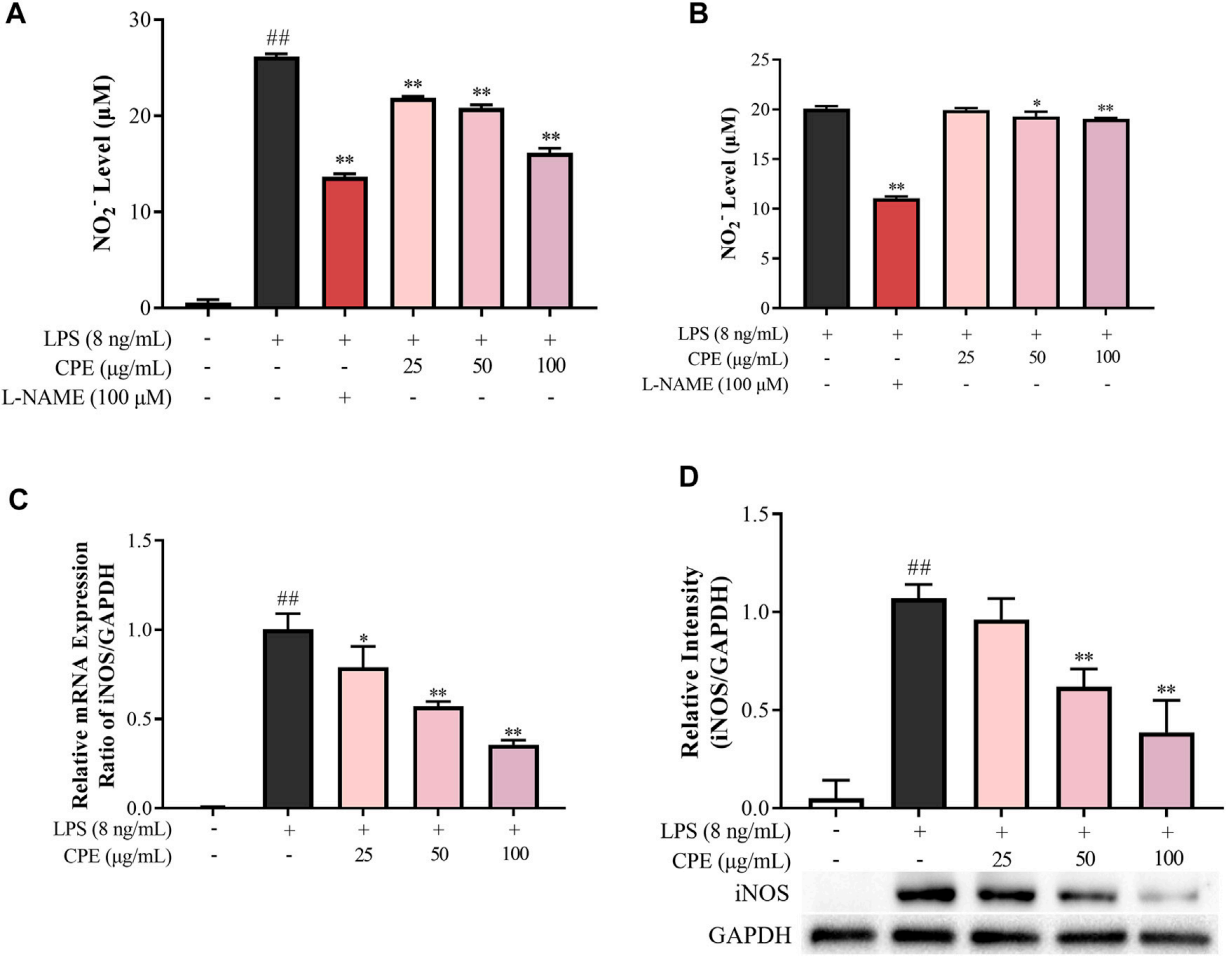
Effect of CPE on LPS-induced NO production in RAW264.7 macrophages (*n* = 3). **(A)** Effect of CPE on supernatant NO in LPS-primed RAW264.7 macrophages. Cells were treated with LPS (8 ng/ml) with or without CPE at the indicated concentrations for 24 h. NO level in the medium was then determined by Griess reagent. L-NAME at 100 μM was used as a positive control. **(B)** Effect of CPE on iNOS activity. RAW264.7 macrophages were treated with LPS (8 ng/ml) for 12 h to induce iNOS. LPS was then removed, and the cells were cultured with the CPE at different concentrations for a further 12 h. Supernatant NO was measured by the Griess reagent to represent the iNOS activity. L-NAME (100 μM) was used as a positive control. **(C)** Effect of CPE on iNOS mRNA expression. RAW264.7 macrophages were cultured with LPS (8 ng/ml) in the absence or presence of CPE for 4 h. Total mRNA was then extracted. The mRNA level of iNOS was determined by RT-qPCR. **(D)** Effect of CPE on iNOS protein level. RAW264.7 macrophages were treated with LPS (8 ng/ml) with or without CPE for 24 h. Total intracellular protein was then extracted and the iNOS protein level was determined by Western blot. ^##^ *p* < 0.01 vs. normal control group; ^*^ *p* < 0.05 and ^**^ *p* < 0.01 vs. LPS alone group.

NO, a small diffusible molecule generated by the inducible enzyme iNOS in activated macrophages, is closely associated with many inflammatory diseases. Our data showed that CPE decreased supernatant NO induced by LPS in a concentration-dependent manner (Figure 5A). iNOS is the prominent synthase of NO in LPS-activated macrophages. Whether its activity or quantity is affected, NO production will be changed. Thus, we next investigated the effect of CPE on iNOS activity. RAW264.7 macrophages were treated with LPS for 12 h and then washed by PBS to remove LPS. In this context, the quantity of iNOS was fixed, and the level of supernatant NO was mainly decided by iNOS activity. The obtained data showed that CPE slightly affected iNOS activity although the positive drug L-NAME could markedly do (Figure 5B). We further determined the effect of CPE on iNOS expression. As expected, CPE potently inhibited LPS-induced iNOS mRNA and protein expression in a concentration-dependent manner (Figures 5C,D). These results indicated that the reduction effect of CPE on supernatant NO was mainly attributed to suppressing iNOS expression rather than its activity.

#### 3.3.2 CPE decreases LPS-induced PGE_2_ production by suppressing COX-2 expression

**FIGURE 6:**
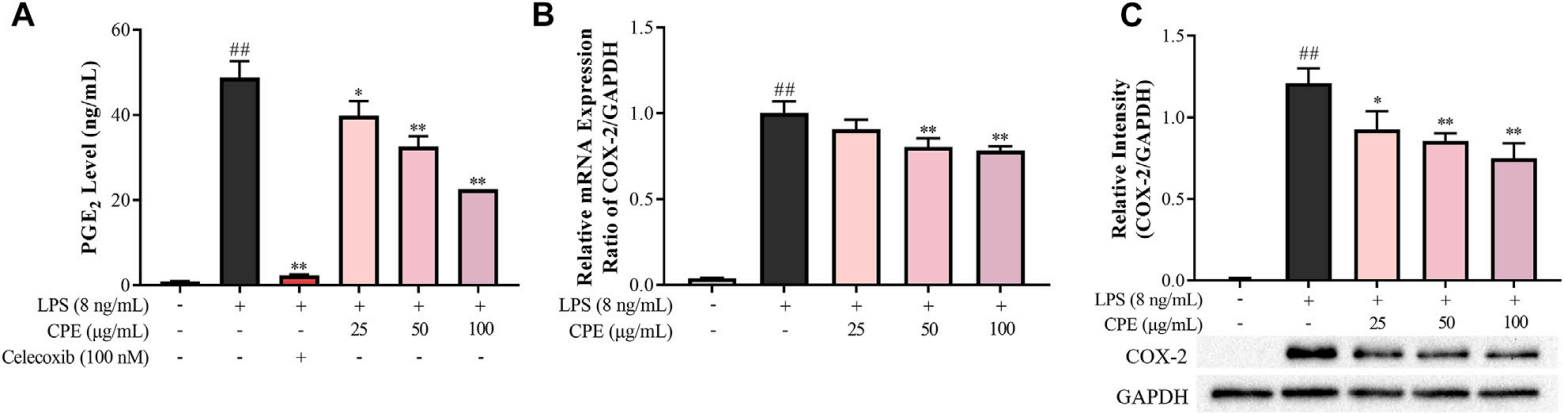
Effect of CPE on PGE_2_ production in LPS-stimulated RAW264.7 macrophages (*n* = 3). **(A)** Effect of CPE on supernatant PGE_2_ in LPS-activated RAW264.7 macrophages. Cells were incubated with LPS (8 ng/ml) with or without CPE for 24 h. PGE_2_ level in the supernatant was then detected by ELISA. Celecoxib was used as a positive control. **(B)** Effect of CPE on COX-2 mRNA expression. RAW264.7 macrophages were treated with LPS (8 ng/ml) with or without CPE at the indicated concentrations for 4 h. Total mRNA was then extracted. The mRNA level of COX-2 was determined by RT-qPCR. **(C)** Effect of CPE on COX-2 protein expression. RAW264.7 macrophages were treated with LPS (8 ng/ml) in the absence or presence of CPE for 24 h. Total intracellular protein was then extracted, and the COX-2 protein level was assayed by Western blot. ^##^ *p* < 0.01 vs. normal control group; ^*^ *p* < 0.05 and ^**^ *p* < 0.01 vs. LPS alone group.

PGE_2_ is another important inflammatory mediator which mediates fever and pain (Kawabata, 2011). Our result showed that CPE could potently inhibit LPS-elevated supernatant PGE_2_ in a concentration-dependent manner (Figure 6A). As we all know, LPS can strongly induce the expression of COX-2, a pivotal rate-limiting enzyme of PGE_2_ synthesis (Bazan, 2001). Therefore, we next investigated the effect of CPE on the expression of COX-2. As presented in Figures 6B,C, CPE could decrease LPS-induced COX-2 mRNA and protein expression. These results indicated that CPE exerted its inhibitory effect on supernatant PGE_2_ by downregulating COX-2 expression.

#### 3.3.3 CPE suppresses the production of IL-6 and IL-1β in LPS-stimulated RAW264.7 macrophages

**FIGURE 7:**
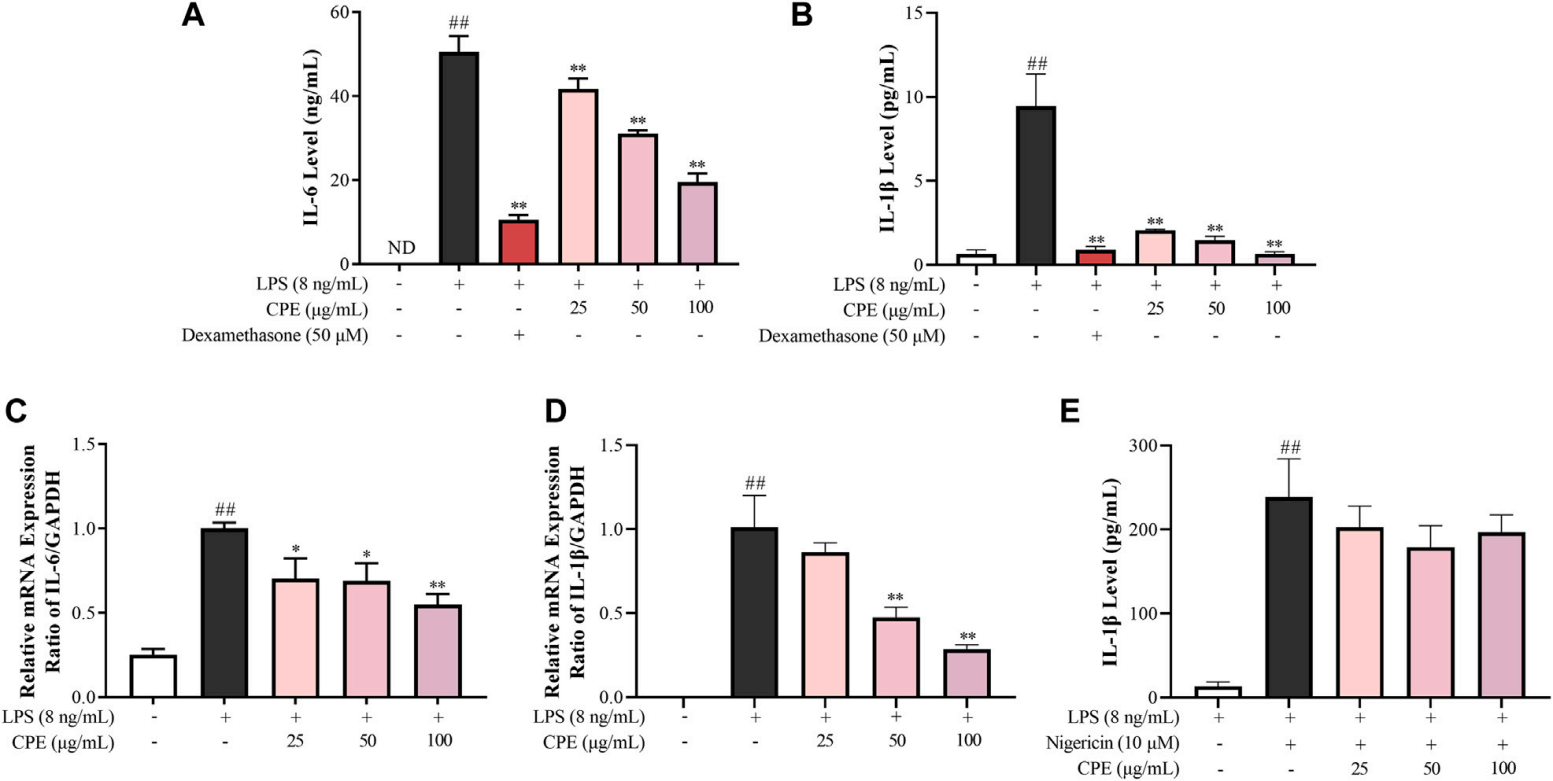
Effects of CPE on IL-6 and IL-1β production in LPS-stimulated RAW264.7 macrophages (*n* = 3). **(A,B)** Effects of CPE on supernatant IL-6 **(A)** and IL-1β **(B)** in LPS-stimulated RAW264.7 macrophages. The cells were treated with LPS (8 ng/ml) with or without CPE for 24 h. IL-6 and IL-1β levels in the supernatant were then detected by ELISA. Dexamethasone was used as a positive control. **(C,D)** Effects of CPE on the mRNA levels of IL-6 **(C)** and IL-1β **(D)** in LPS-stimulated RAW264.7 macrophages. The cells were exposed to LPS (8 ng/ml) and incubated with or without designated concentrations of CPE for 4 h. Total RNA was then extracted and reverse transcribed, and the mRNA expressions of IL-6 and IL-1β were determined by RT-qPCR. **(E)** Effect of CPE on NLRP3 inflammasome activity in BMDMs. BMDMs were primed with LPS (8 ng/ml) for 12 h and then treated with different concentrations of CPE for 30 min. Then, the cells were treated with nigericin (10 µM) for further 45 min to activate NLRP3 inflammasome. Supernatant IL-1β level was detected by ELISA. ^##^ *p* < 0.01 vs. normal control group; ^*^ *p* < 0.05 and ^**^ *p* < 0.01 vs. LPS alone group. ND, not detected.

IL-6 and IL-1β, the prototypic pro-inflammatory cytokines, play crucial roles in acute and chronic inflammations and autoimmune disorders (Ren and Torres, 2009; Tanaka et al., 2014). Our data showed that CPE concentration-dependently reduced supernatant IL-6 and IL-1β in LPS-activated RAW264.7 macrophages (Figures 7A,B). RT-qPCR assay was then performed to determine the effects of CPE on the mRNA levels of IL-6 and IL-1β. Consistently, CPE downregulated IL-6 and IL-1β transcriptions in LPS-stimulated RAW264.7 macrophages (Figures 7C,D).

To our knowledge, the secretion of mature IL-1β is also correlated with the degree of NLRP3 inflammasome activation (Garcia et al., 2016). Thus, we next investigated whether CPE could affect the activity of NLRP3 inflammasome. As shown in Figure 7E, nigericin could increase the supernatant IL-1β almost 18-fold by activating NLRP3 inflammasome, while CPE had no obvious effect on nigericin-induced NLRP3 inflammasome activation (*p* > 0.05), indicating that the inhibitory effect of CPE on supernatant IL-1β should be attributed to suppressing its expression, rather than inactivating NLRP3 inflammasome.

#### 3.3.4 CPE inhibits LPS-activated NF-κB signaling through impeding the phosphorylation and degradation of inhibitors of NF-κB and translocation of p65

**FIGURE 8:**
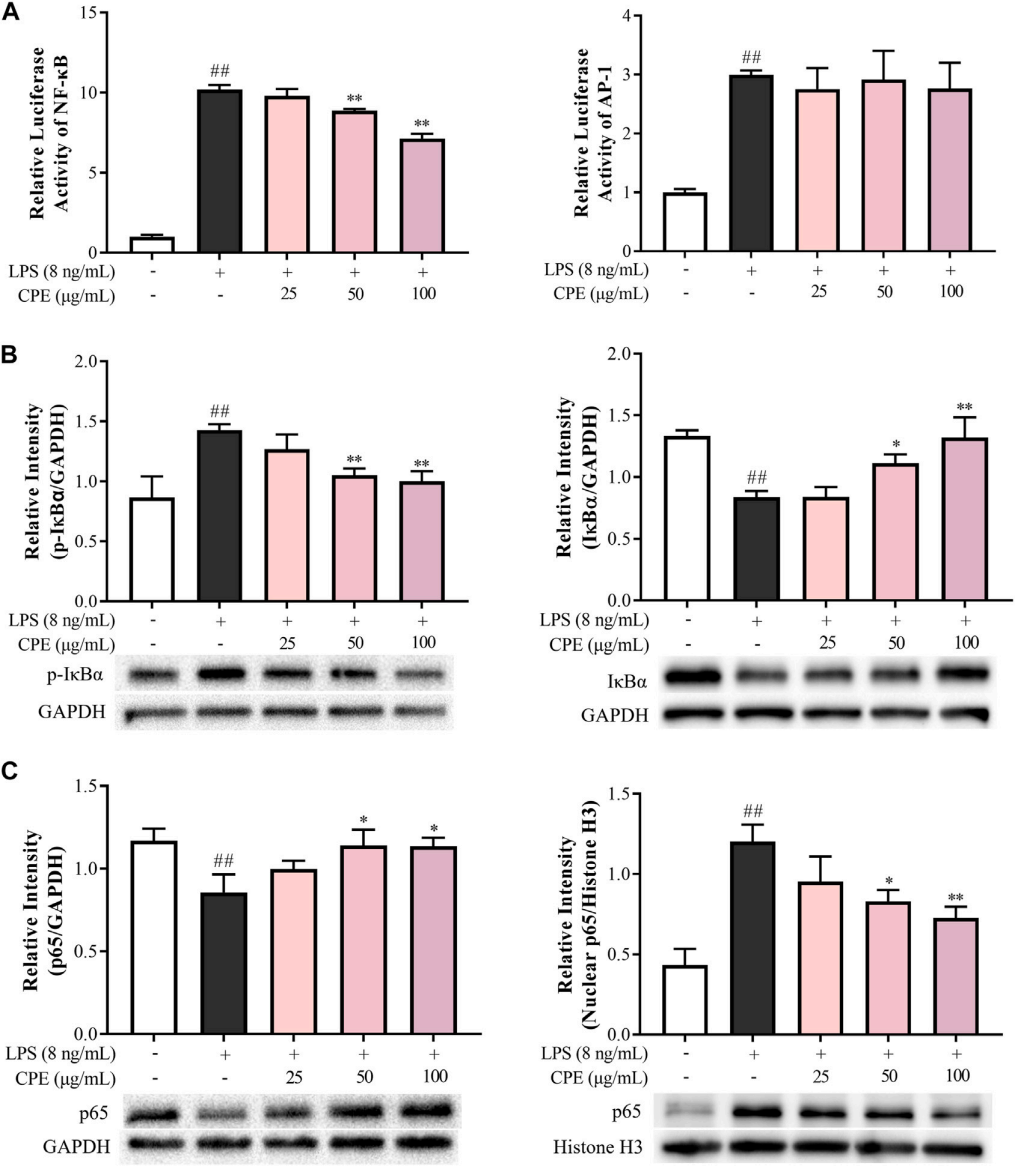
CPE inhibits LPS-activated NF-κB rather than AP-1 signal in RAW264.7 macrophages (*n* = 3). **(A)** Effects of CPE on LPS-activated NF-κB and AP-1. The cells transfected with pNFκB-TA-luc or pAP1-TA-luc plasmid were treated with LPS (8 ng/ml) with or without CPE for 4 h. The luciferase activities were determined. **(B,C)** Effects of CPE on LPS-induced IκBα phosphorylation and degradation, and p65 translocation from the cytoplasm to the nucleus. RAW264.7 macrophages were treated with LPS (8 ng/ml) with or without CPE for 30 min. Cytoplasmic and nuclear proteins were then extracted separately for Western blot. ^##^ *p* < 0.01 vs. normal control group; ^*^ *p* < 0.05 and ^**^ *p* < 0.01 vs. LPS alone group.

As we know, the above inflammatory genes can be regulated by NF-κB and AP-1 signaling (Kawasaki and Kawai, 2014). To investigate the upstream mechanism for the anti-inflammatory effects of CPE, luciferase reporter assays of NF-κB and AP-1 were performed. As shown in Figure 8A, LPS markedly promoted the activations of NF-κB and AP-1, while CPE concentration-dependently suppressed LPS-caused NF-κB activation but little affected AP-1 signaling. Further, CPE not only inhibited the phosphorylation and degradation of IκBα (Figure 8B), but also impeded the nuclear translocation of p65 (Figure 8C). These findings demonstrated that CPE was mainly through inhibiting NF-κB signaling to suppress the transcription of pro-inflammatory genes.

#### 3.3.5 CPE alleviates endotoxemia in mice

**FIGURE 9:**
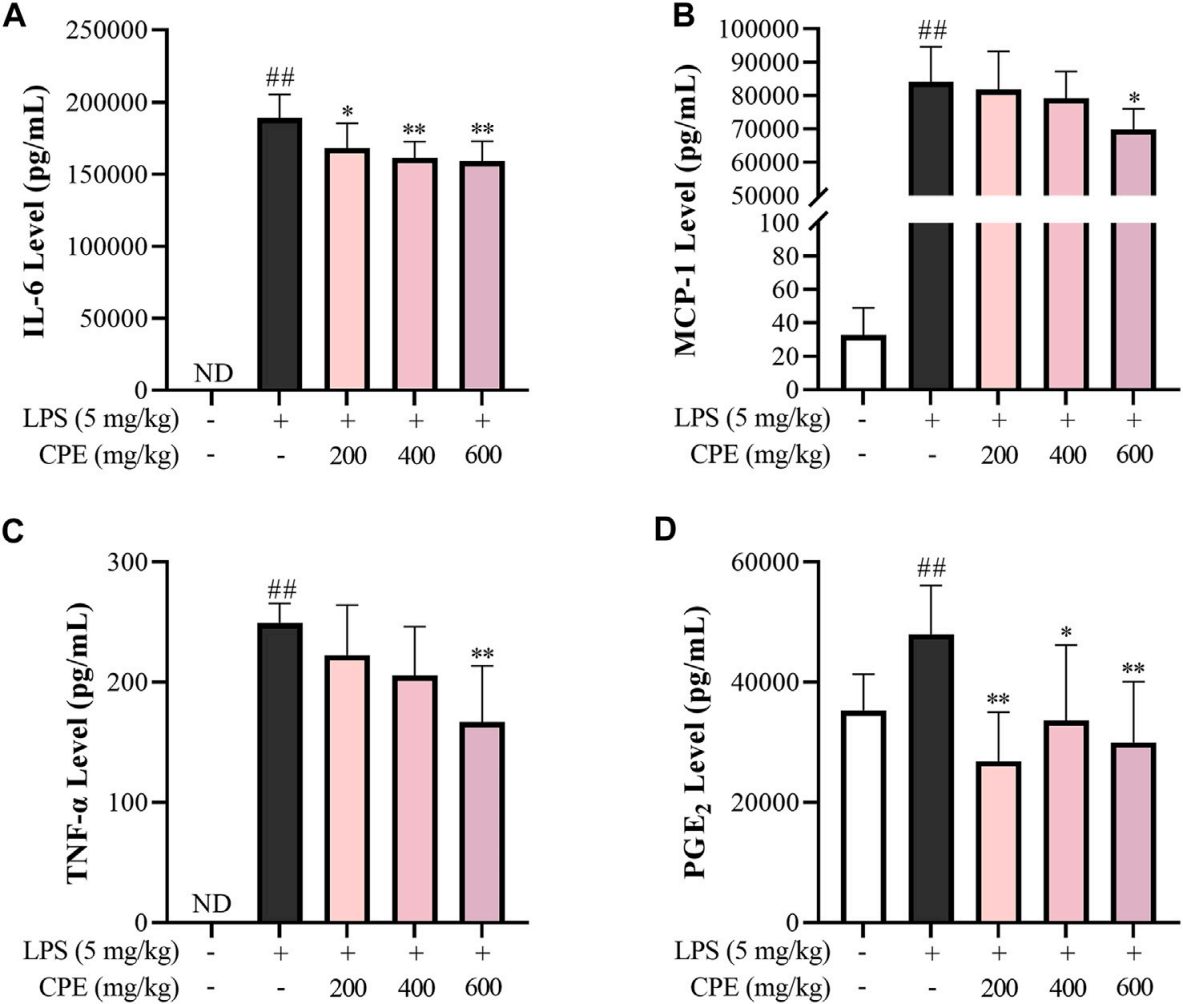
Effect of CPE on endotoxemia mice (*n* = 8). The male C57BL/6 mice were administrated CPE (*i.p.*) immediately after LPS injection (*i.v.*). 2 h later, the blood was collected. The levels of IL-6 **(A)**, MCP-1 **(B)**, TNF-α **(C)**, and PGE_2_ **(D)** in serum were measured by ELISA. ^##^ *p* < 0.01 vs. normal control group; ^*^ *p* < 0.05 and ^**^ *p* < 0.01 vs. model group. ND, not detected.

The mouse endotoxemia model was used for evaluating the *in vivo* anti-inflammatory effect of CPE. As shown in Figure 9, serum IL-6, MCP-1, TNF-α, and PGE_2_ levels dramatically increased 2 h after LPS injection, while CPE (200 mg/kg–600 mg/kg) could suppress them to varying degrees in a dose-dependent manner.

## 4 Discussion

The tepals of wintersweet flower are waxy in nature and the overall color of the flower is yellow. Its essential oil or other products are widely used in perfumes, cosmetics, and aromatherapy (Aslam et al., 2020). Previous phytochemical studies have revealed that the principal pigments in tepals are the metabolite of flavonoids, such as quercetin and rutin (Yang et al., 2018). In the present study, our results showed that total flavonoids in CPE were 7.980% ± 0.176%, of which the contents of rutin and quercetin were 4.158% ± 0.094% and 0.128% ± 0.006%, respectively (Figure 2B). Among multiple biological activities of flavonoids, antioxidation is predominant (Pietta, 2000). Indeed, CPE possessed potent ferric reducing ability (Figure 3A), and scavenging activity for DPPH radical and superoxide anion (Figures 3B,C) in cell-free systems. In cell system, it could reduce ROS production by inactivating NADPH oxidase (Figures 4B,D).

In addition to antioxidation, flavonoids often exhibit anti-inflammatory actions (Maleki et al., 2019), which are also the usual bioactivity of alkaloids (Bai et al., 2021). Previous studies have reported that alkaloids can exist in various parts of wintersweet including its flowers (Shu et al., 2019). Indeed, our study showed that the total alkaloids content in CPE was 1.461% ± 0.041%, of which two characteristic components—calycanthine and chimonanthine were identified with the content of 0.047% ± 0.002% and 0.708% ± 0.015%, respectively (Figure 2A). In endotoxemia mice, CPE could decrease IL-6, MCP-1, TNF-α, and PGE_2_ levels in the serum after a single therapeutic administration (Figure 9), showing a definitely anti-inflammatory activity.

As a major effector cell of LPS, macrophage mediates non-specific immune responses against pathogens and plays a crucial role in the occurrence and progression of inflammation by virtue of releasing massive cytokines (Franken et al., 2016). Therefore, we next investigated the anti-inflammatory action of CPE in LPS-stimulated macrophages, which is one of the most commonly used *in vitro* inflammatory models. NO and PGE_2_, two small molecules derived from enzyme reactions, are involved in numerous physiological and pathological processes (Thomas et al., 2008; Zhou et al., 2012). Excess NO can cause vasodilatation, cell apoptosis, and tissue destruction (Han et al., 2021). And overproduction of PGE_2_ may result in fever, pain, increased vascular permeability, edema formation, and immune cell infiltration (Hung et al., 2019). In LPS-activated macrophages, their key synthases—iNOS and COX-2—are induced to express and subsequently catalyze the generation of NO and PGE_2_, respectively (Zamora et al., 2000; Hegde et al., 2011). Our results showed that CPE could significantly suppress the expressions of iNOS and COX-2 at both transcriptional and translational levels (Figures 5C,D, 6B,C), thus consequently decreasing supernatant NO and PGE_2_ (Figures 5A, 6A), respectively.

As the endogenous pyrogens, both IL-6 and IL-1β play important roles in acute inflammation as well as febrile responses (Kozak et al., 1998). IL-6, promptly generated in response to infections and tissue injuries, is a potent inducer of acute phase response that triggers the production of a broad range of acute phase proteins, such as C-reactive protein and complement C3 (Tanaka et al., 2018). IL-1β not only participates in the activation and recruitment of inflammatory cells, but also directly induces the release of inflammatory mediators (Gabay et al., 2010). CPE could downregulate the mRNA transcriptions of IL-6 and IL-1β and subsequently lower their levels in the supernatant of LPS-activated RAW264.7 macrophages (Figures 7A–D). Different from IL-6, IL-1β exists in the cytoplasm as an inactive precursor form (pro-IL-1β) which can be cleaved into mature and bioactive form by activated NLRP3 inflammasome (Swanson et al., 2019). Therefore, we also determined the effect of CPE on NLRP3 inflammasome activation and mtROS, a direct activator of NLRP3 (Iyer et al., 2013; Swanson et al., 2019). However, neither mtROS level (Figure 4C) nor NLRP3 inflammasome activity could be affected by CPE (Figure 7E). Our findings indicated that CPE decreased IL-6 and IL-1β in the supernatant mainly through suppressing their transcriptions.

The transcriptions of above-mentioned inflammatory cytokines are known to be regulated by NF-κB, a rapid-acting transcription factor in response to multiple cellular stimuli (Pateras et al., 2014). Upon LPS-stimulation, IKK-β is activated and thereby triggers the phosphorylation and ubiquitin-dependent degradation of IκBα, resulting in the rapid and transient nuclear translocation of p50/p65 dimers which bind to a specific DNA element to mediate transcription of target inflammatory genes (Liu et al., 2017). Indeed, CPE could inhibit the phosphorylation and degradation of IκBα, prevent the nuclear translocation of p65, thus dampening LPS-activated NF-κB signaling (Figure 8).

## 5 Conclusion

Our study indicated, for the first time, that CPE possesses both anti-inflammatory and anti-oxidative properties. *In vitro*, it decreases LPS-elevated pro-inflammatory mediators in macrophages through suppressing the phosphorylation and degradation of IκBα thus restraining the nuclear translocation of p65 and dampening NF-κB signaling. *In vivo*, it reduces the levels of serum pro-inflammatory cytokines in endotoxemia mice. Moreover, CPE also exerts markedly anti-oxidative effects in cell systems and cell-free systems. Our findings provide new insight into the functions of wintersweet flower, which may broaden its applications and be beneficial to developing its natural health products.

## Data Availability

The original contributions presented in the study are included in the article/Supplementary Material, further inquiries can be directed to the corresponding authors.

## Abbreviations

BMDMs, bone marrow-derived macrophages; COX-2, cyclooxygenase-2; CPE, ethanol extract of Chimonanthi Praecocis Flos; DPBS, Dulbecco’s phosphate-buffered saline; IKK, IκB kinase; IL-1β, interleukin-1β; IL-6, interleukin-6; iNOS, inducible nitric oxide synthase; L-NAME, L-N^G^-Nitro arginine methyl ester; LPS, lipopolysaccharide; M-CSF, macrophage colony-stimulating factor; mtROS, mitochondrial ROS; NLRP3, NLR family pyrin domain containing 3; OD, optical density; PGE_2_, prostaglandin E_2_; RNS, reactive nitrogen species; ROS, reactive oxygen species; RT-qPCR, quantitative real-time PCR; TLR4, Toll-like receptor 4
