# Supplementary material for: Anti-oxidative and anti-inflammatory activities of the ethanol extract of edible flower from Chimonanthus praecox
Source: Front Pharmacol. 2022 Sep 27;13:1004520. doi: 10.3389/fphar.2022.1004520 (PMC9552299; doi:10.3389/fphar.2022.1004520)

Supplementary file of raw WB images

Fig. 5D

Repeat 1

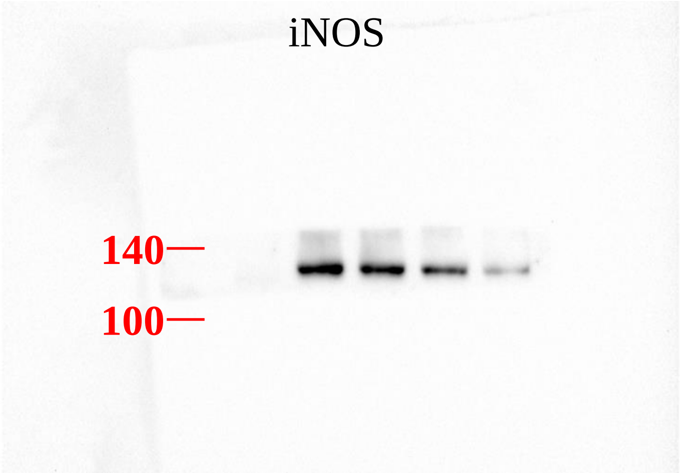

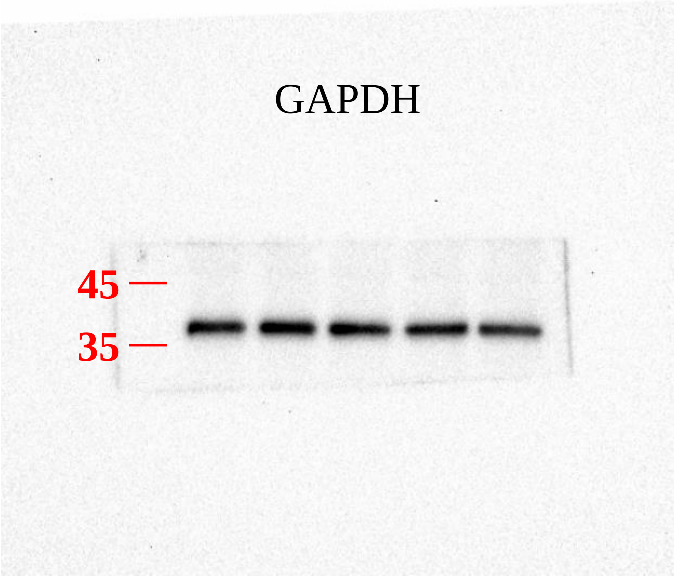

Repeat 2

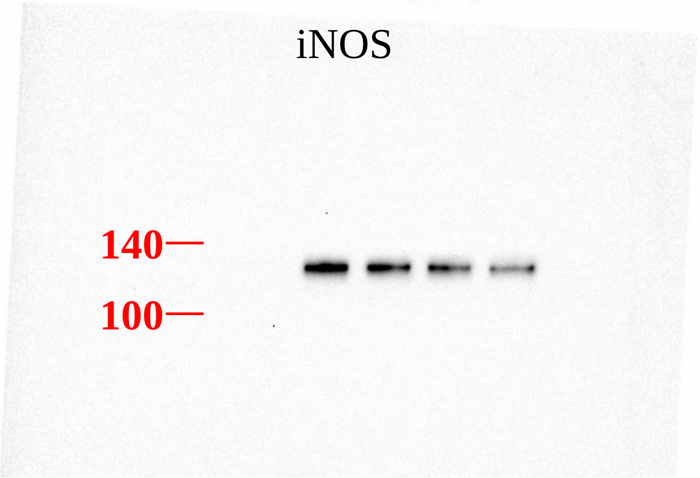

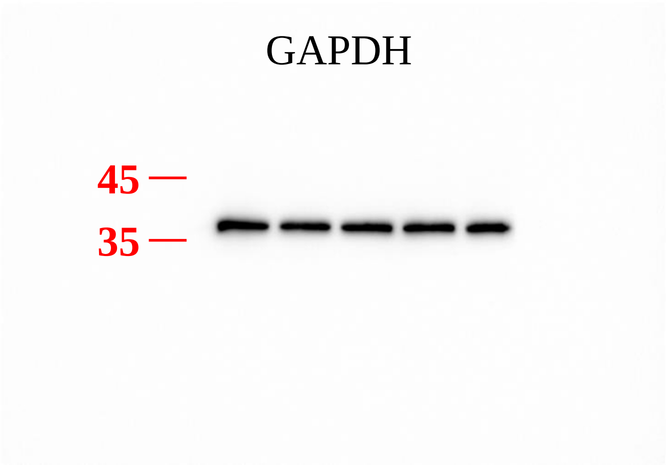

Repeat 3

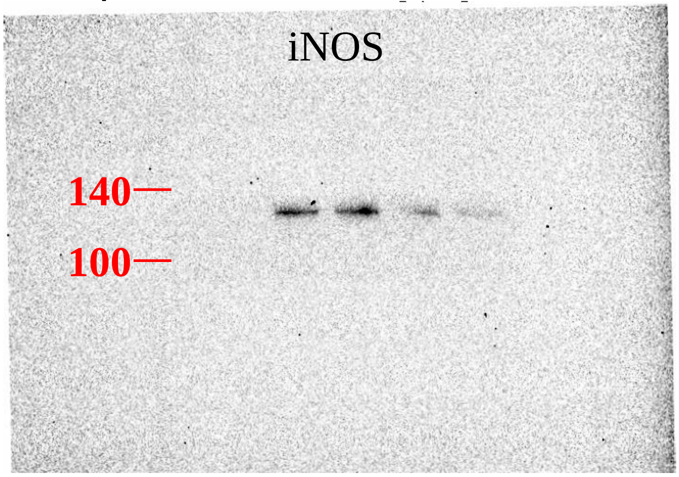

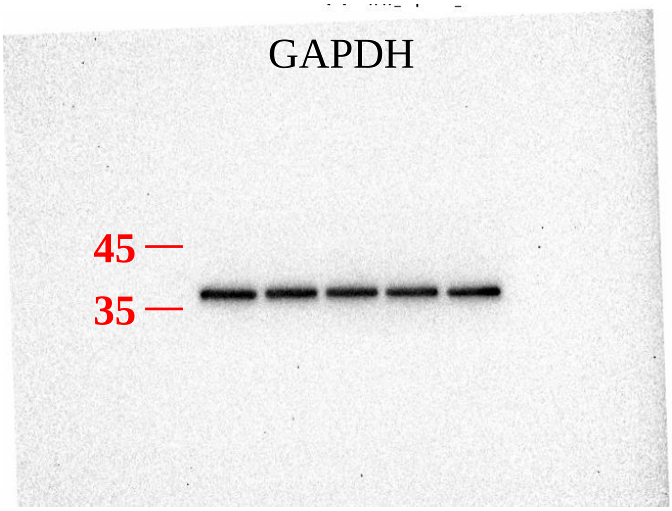

Fig. 6C

Repeat 1

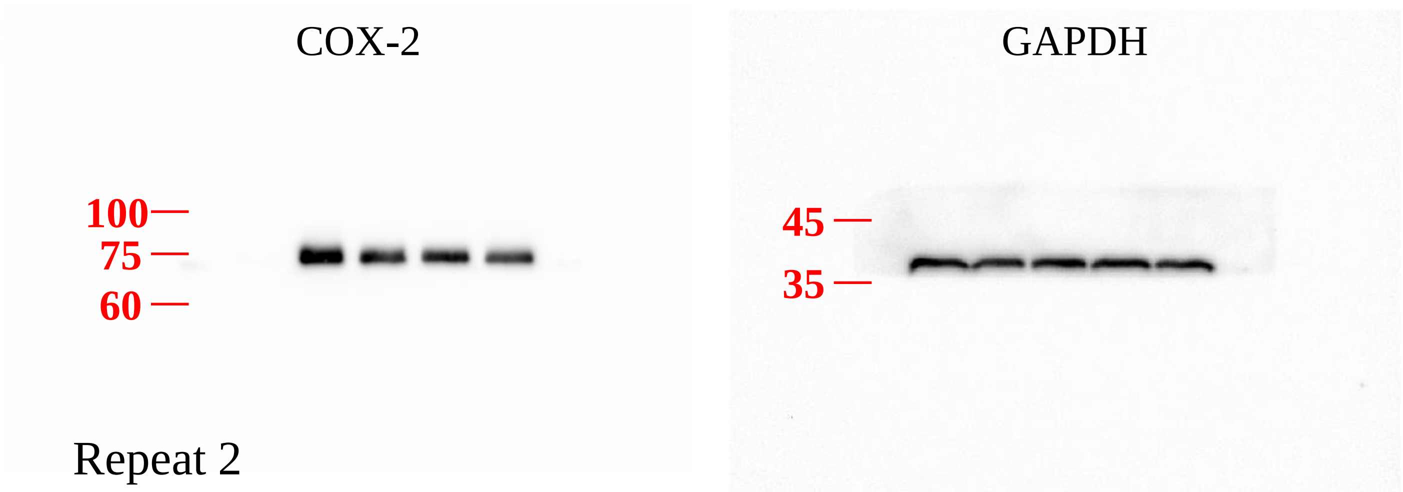

Repeat 2

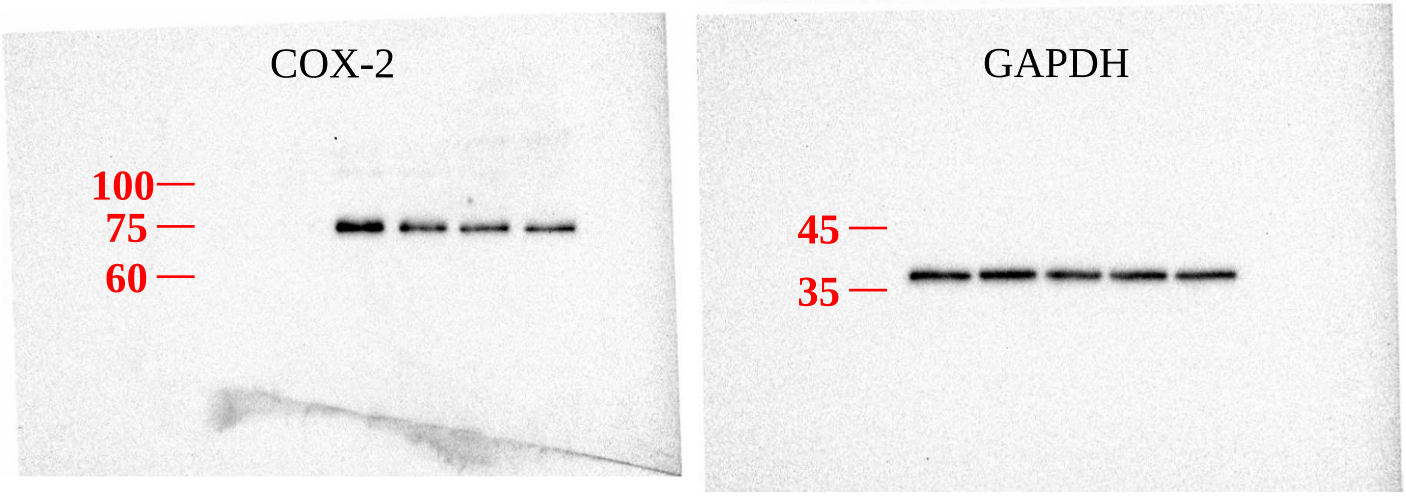

Repeat 3

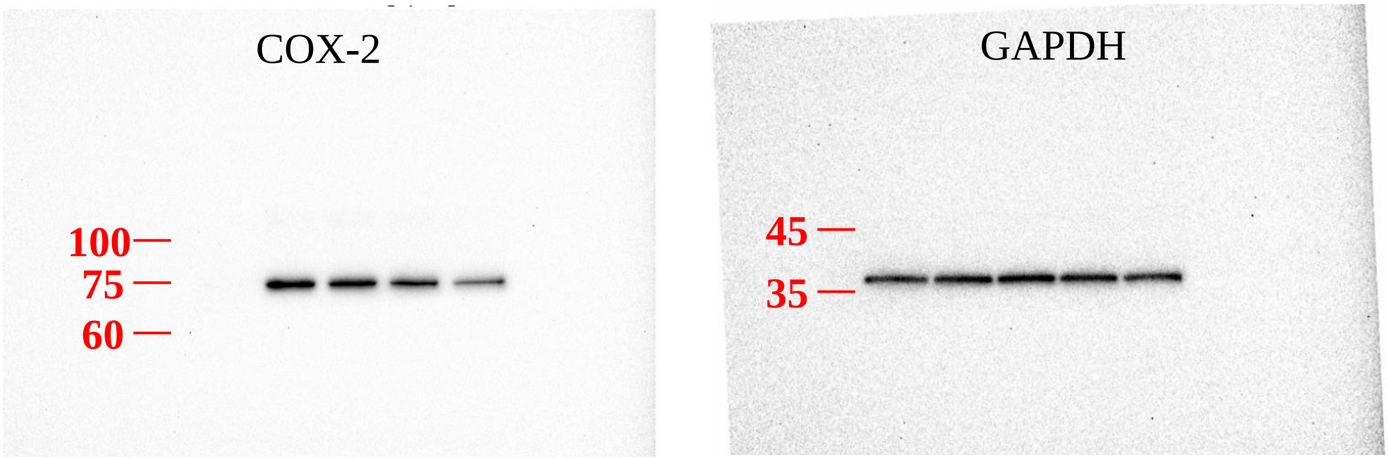

Fig. 8B

Repeat 1

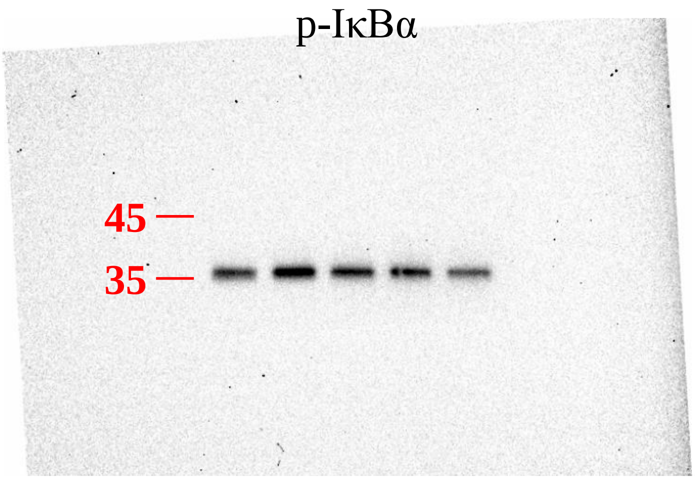

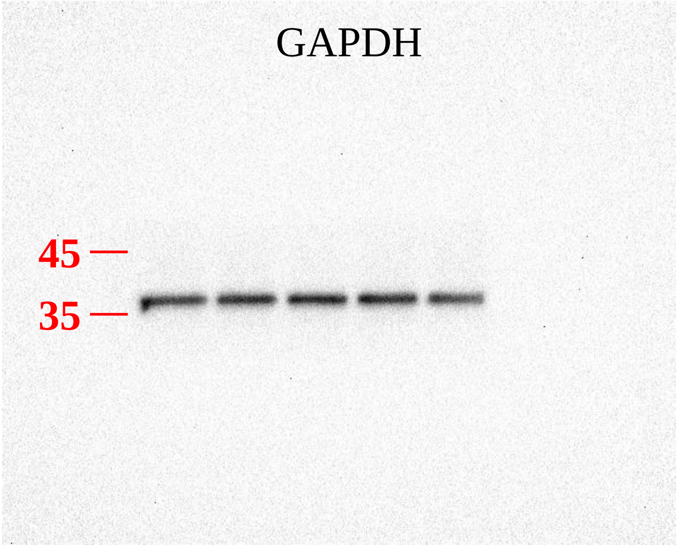

Repeat 2

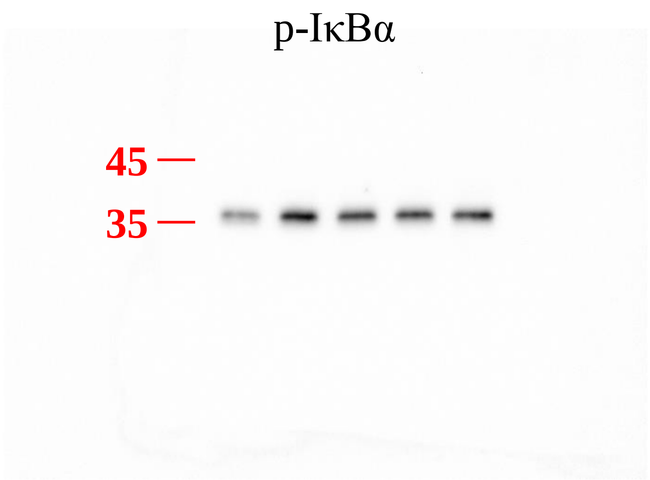

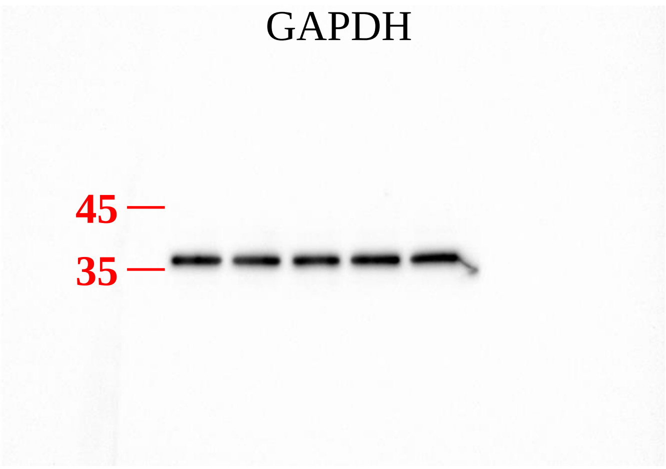

Repeat 3

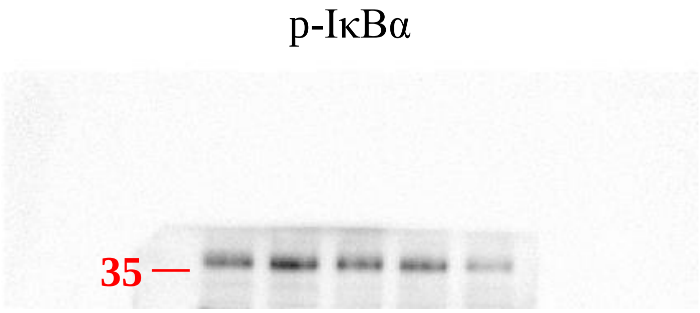

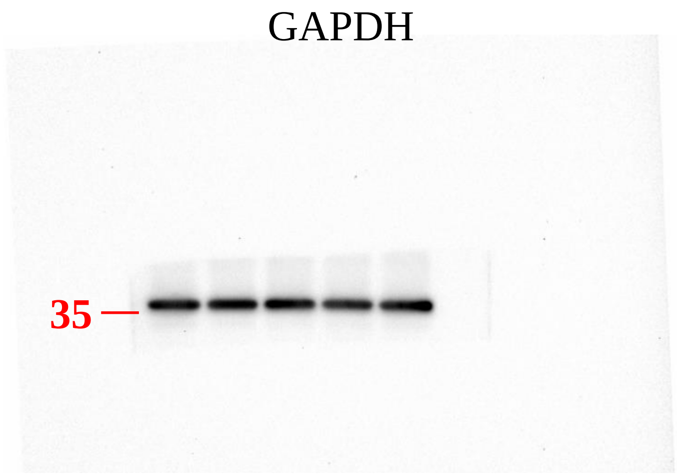

Fig. 8B

Repeat 1

IκBα

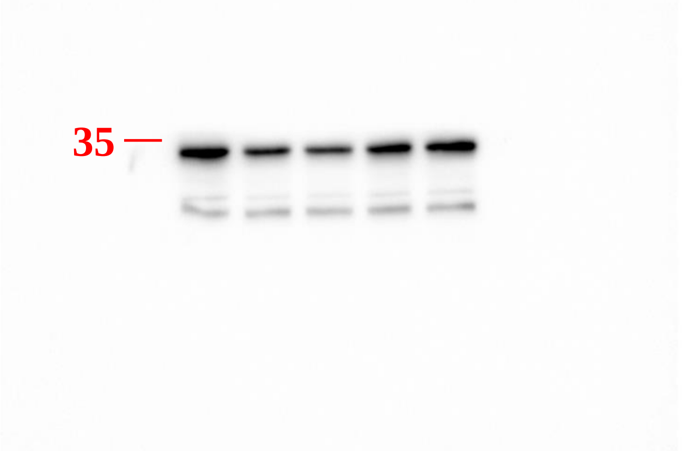

GAPDH

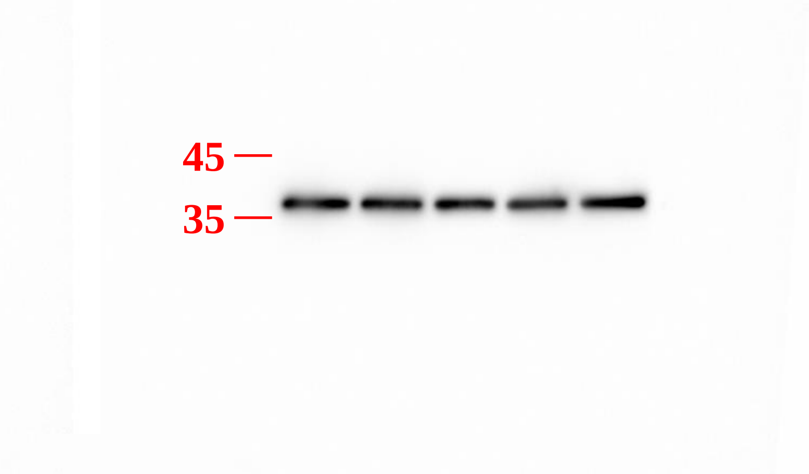

Repeat 2

IκBα

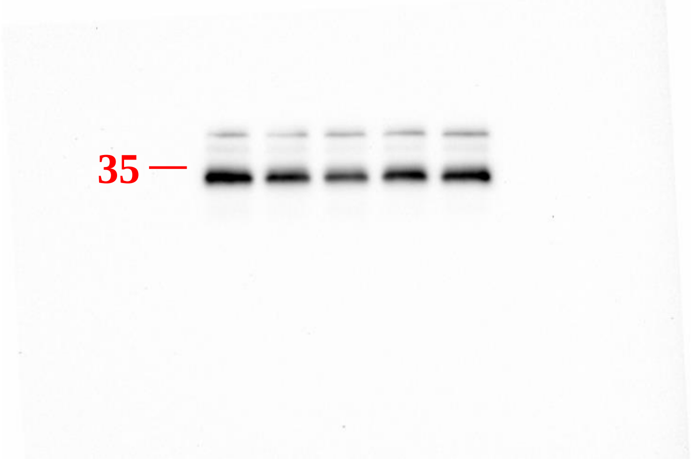

GAPDH

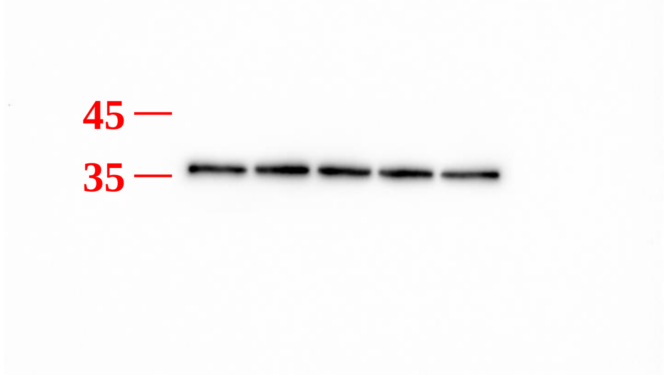

Repeat 3

IκBα

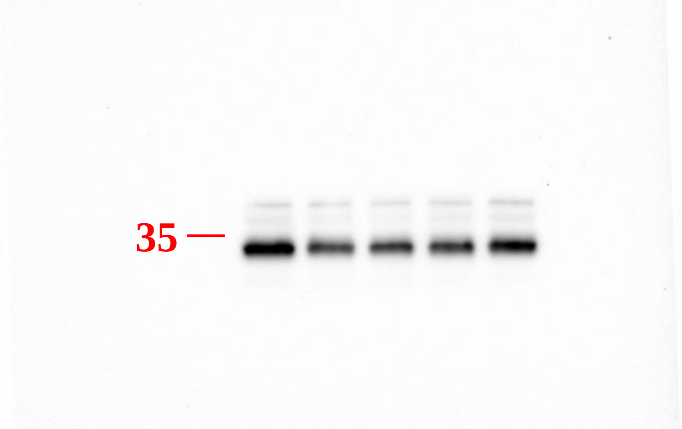

GAPDH

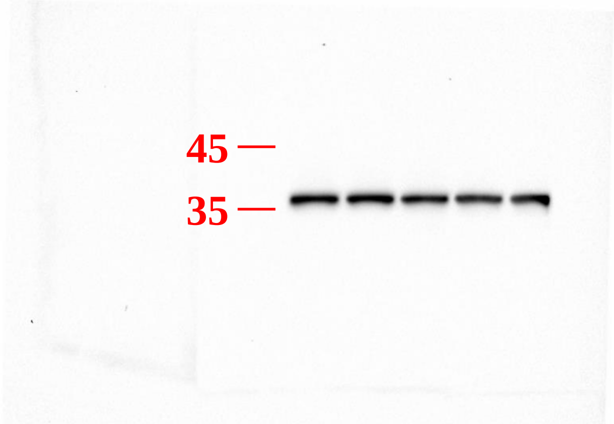

Fig. 8C

Repeat 1

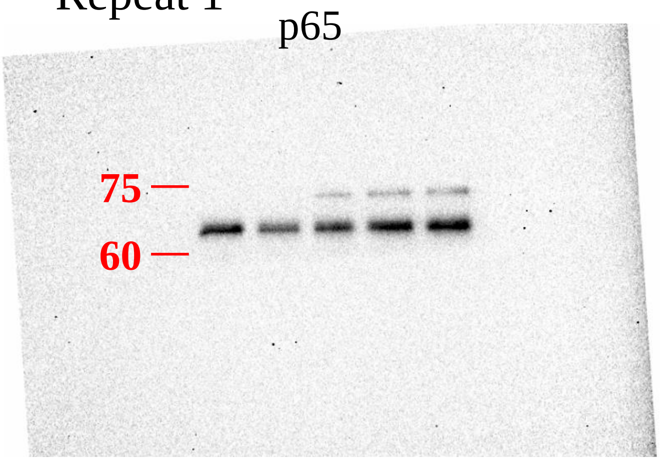

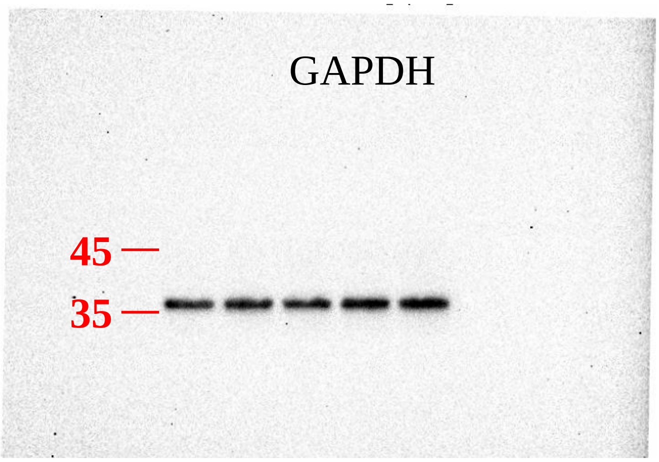

Repeat 2

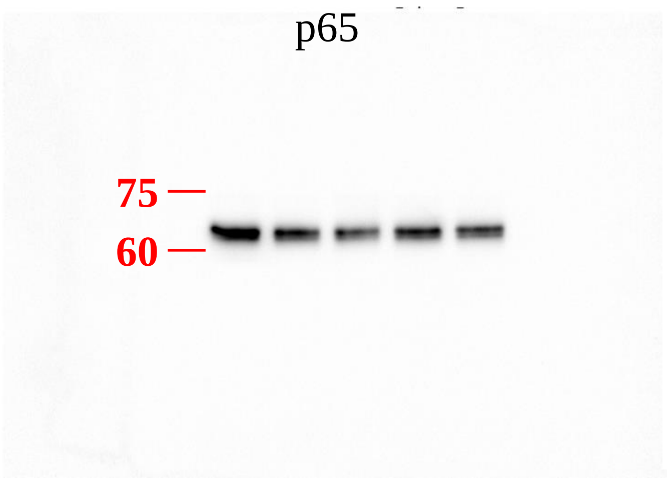

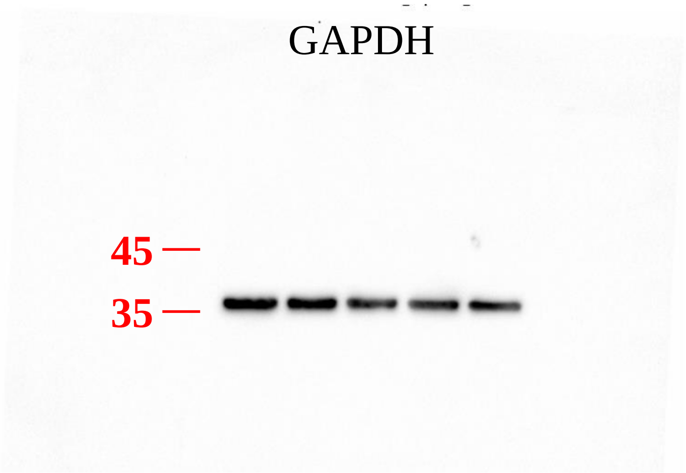

Repeat 3

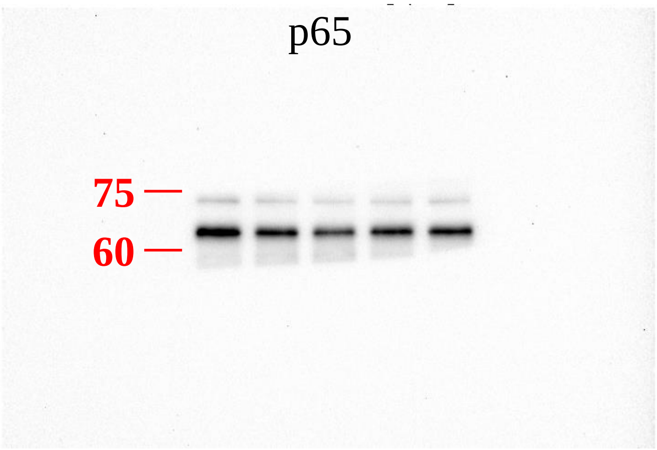

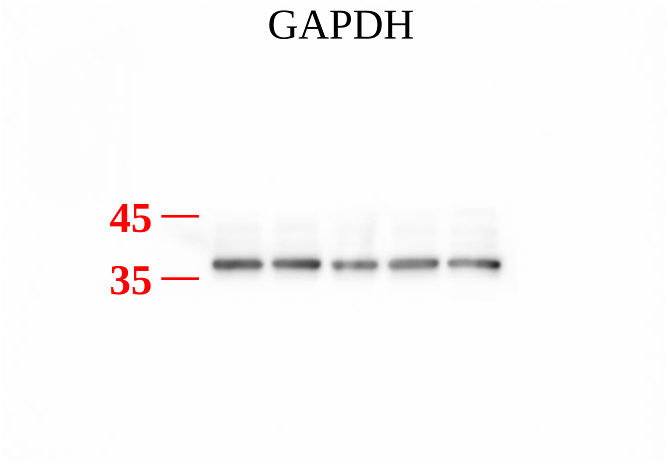

Fig. 8C

Repeat 1

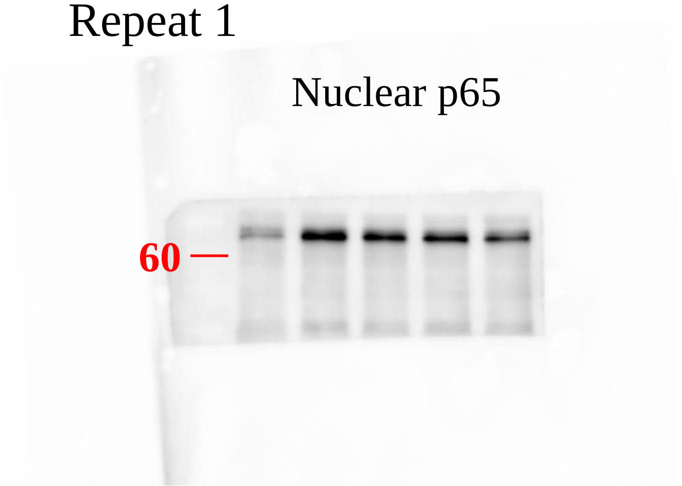

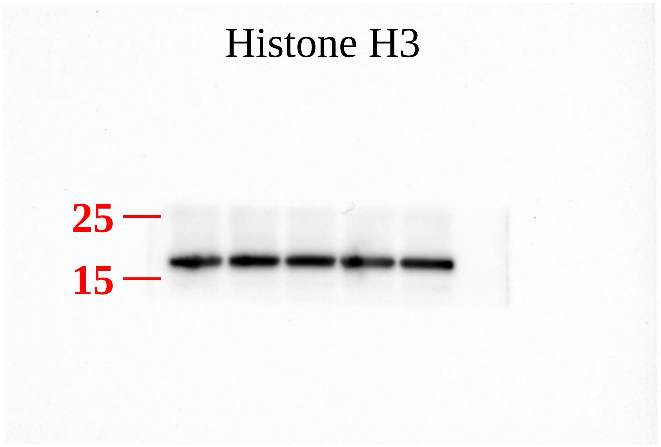

Repeat 2

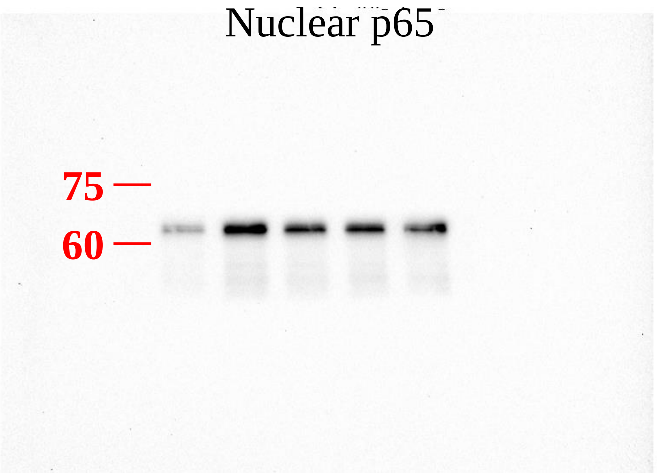

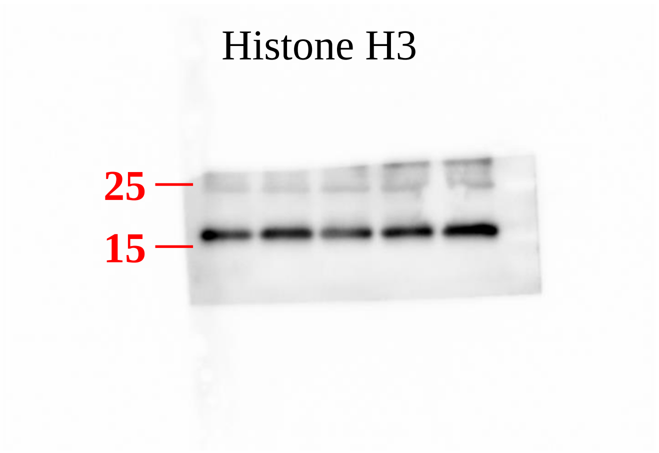

Repeat 3

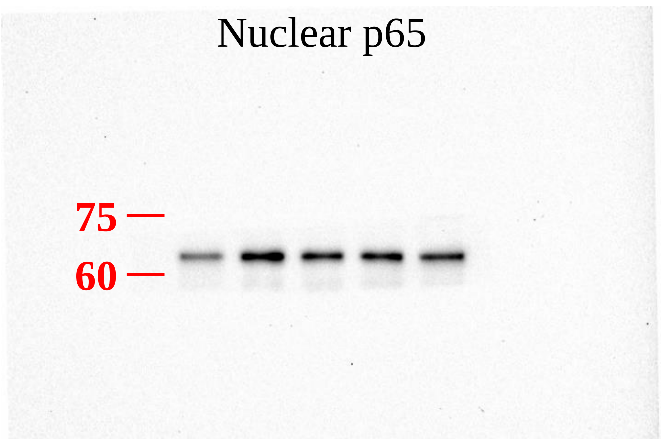

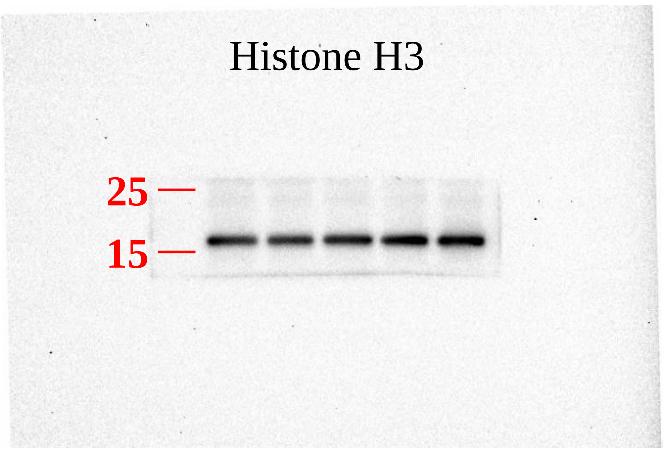

Supplement: Supplementary file 4 [file DataSheet1.PDF]
